# 室内灰尘中氟化液晶单体的赋存特征及人体健康暴露风险

**DOI:** 10.3724/SP.J.1123.2025.03021

**Published:** 2025-10-08

**Authors:** Shiyuan WANG, Kai HUA, Jiahao HE, Ke GAO, Liping LU

**Affiliations:** 北京工业大学环境科学系，区域大气复合污染防治北京市重点实验室，北京 100124; Department of Environmental Science，Beijing University of Technology，Key Laboratory of Beijing on Regional Air Pollution Control，Beijing 100124，China

**Keywords:** 氟化液晶单体, 全二维气相色谱-飞行时间质谱, 室内灰尘, 人群差异, 健康风险评估, fluorinated liquid crystal monomers （FLCMs）, full two-dimensional gas chromatography-time-of-flight mass spectrometry （GC×GC-TOF MS）, indoor dust, population differences, health risk assessment

## Abstract

氟化液晶单体（FLCMs）作为液晶显示器（LCD）的关键材料，可在其生命周期中释放到环境，被视为新型持久性有机污染物。室内灰尘中的FLCMs污染与人体健康紧密相关，但其赋存特征及人群健康风险差异尚不明确。本研究利用全二维气相色谱-飞行时间质谱（GC×GC-TOF MS）分析了实验室、宿舍、教室、打印店和食堂5种室内灰尘中FLCMs的赋存特征，并采用蒙特卡洛模拟评估人群差异化健康风险。结果显示，20种FLCMs的总含量范围为3.68~593 ng/g（中位数85.1 ng/g），其中打印店含量最高（206~593 ng/g），其次为实验室（89.0~219 ng/g）、教室（28.4~137 ng/g）。这种分布差异与电子设备数量/种类、通风条件及清洁频率密切相关。健康风险评估表明，4-［二氟（3，4，5-三氟苯氧基）甲基］-3，5-二氟-4′-丙基联苯（tFPO-CF2-dF3B）和4-［反-4-（反-4-乙基环己基）环己基］-1-三氟甲氧基苯（2bcHtFMeOP）的健康风险熵（HQ，>1.00×10^-6^）高于其他FLCMs，这归因于其较高浓度和自身毒性。打印店对各年龄段人群的危害指数（HI）最高（HI中位数范围：1.88×10^-5^~1.60×10^-4^），为其他场所的2~6倍。差异性分析表明，不同室内场景和不同人群的非致癌风险存在差异，高校主要群体（18~60岁）中女性的非致癌风险更高。本研究弥补了室内灰尘中FLCMs赋存特征及人群差异化健康风险评估的不足，为FLCMs污染的管控和治理提供了全面精细的数据支持。

## Abstract (en)

Fluorinated liquid crystal monomers （FLCMs） are the most widely used materials in liquid crystal display （LCD） manufacturing. They can be released into environmental media during production， use， and disposal， and are recognized as a class of novel persistent organic pollutants. Contamination of FLCMs in indoor dust is closely linked to human health； however， existing research has yet to fully elucidate their occurrence characteristics and population-differentiated health risks within this medium. This study analyzed the occurrence characteristics of FLCMs in dust from five indoor environments （laboratories， dormitories， classrooms， print shops， and cafeterias） using comprehensive two-dimensional gas chromatography coupled with time-of-flight mass spectrometry （GC×GC-TOF MS）. Population-differentiated health risks were further evaluated by Monte Carlo simulation. The results showed that the total contents of 20 target FLCMs ranged from 3.68 to 593 ng/g （median： 85.1 ng/g）. Print shops exhibited the highest ∑FLCMs contents （206-593 ng/g）， followed by laboratories （89.0-219 ng/g） and classrooms （28.4–137 ng/g）. This differential distribution was closely associated with the number and types of electronic devices， ventilation conditions， and indoor cleaning frequency. The health risk assessment indicated that 4-［difluoro（3，4，5-trifluorophenoxy）methyl］-3，5-difluoro-4′-propylbiphenyl （tFPO-CF2-dF3B）， and 4-［*trans*-4-（*trans*-4-ethylcyclohexyl）cyclohexyl］-1-trifluoromethoxybenzene （2bcHtFMeOP） pose a higher health risk （hazard quotient （HQ）>1.00×10^-6^） than the other FLCMs， which is probably result of their higher fugitive contents as well as higher autotoxicity. Print stores had the highest hazard index （HI） for all age groups （median HI range： 1.88×10^-5^-1.60×10^-4^）， which was 2-6 times higher than other sites. Population difference analysis reveals that minors （especially infants and young children） are more sensitive to indoor dust FLCMs exposure， which may be due to the high exposure rate of infants and young children caused by their crawling， playing， and hand mouth contact on the ground. In addition， the results indicate that the estimated daily intakes （EDIs） of elderly people （>60 years old） is higher than that of the 18-45 and 45-60 age groups， which may be related to their longer indoor exposure time and lower cleaning frequency due to their limited mobility. It is worth noting that in the college environment group （18-45 years old）， although the exposure risk in this age group is lower than that of the sensitive population mentioned above， the related health risks cannot be ignored. In addition， it was found that the exposure risk of women in this group was slightly higher than that of men， which may be because women usually spend more time indoors and have a higher frequency of exposure. This study fills the gaps in the occurrence characteristics of FLCMs in indoor dust and population differentiated health risk assessment， providing comprehensive and detailed data support for the control and management of FLCMs pollution.

液晶显示器（liquid crystal display，LCD）广泛用于手机、笔记本电脑和液晶电视等电子产品^［1］^。2019年，全球的LCD共产生了670万吨电子垃圾，2023年，来自LCD的电子垃圾为930万吨，占电子垃圾总质量的12.5%^［2］^。液晶单体（liquid crystal monomers， LCMs）是LCD的关键材料，其在液晶面板中仅通过物理吸附填充于偏振片间隙，未与玻璃基板或电极形成化学键合，因此容易释放到环境中^［3］^。目前商业化的LCMs有数千种，种类繁多。通常LCMs具有联苯或环己烷的结构单元，并带有酯、炔烃、氰基、硝基和氟等不同取代基团^［2］^。含氟取代基团的氟化液晶单体（fluorinated LCMs，FLCMs）因其具有低驱动电压、高热稳定性、低黏度及优异的光电性能，已成为应用最广泛的LCMs^［4］^。随着科技的快速发展，电子产品迅速更新换代，导致越来越多的LCD成为电子垃圾^［5］^，使更多的FLCMs进入环境。FLCMs潜在的环境污染和人体健康风险已引起科学界的关注。

FLCMs已在空气^［6］^、沉积物^［7］^、土壤^［8］^、垃圾渗滤液^［9］^和灰尘^［10］^等基质中检测到。灰尘是室内环境中FLCMs主要的赋存介质。Cheng等^［11］^在电子垃圾回收厂的灰尘中检测到了32种FLCMs。Zhu等^［1］^在LCD拆卸车间的灰尘中检出34种FLCMs，总含量为225~97 600 ng/g。Yang等^［12］^在北京收集的室内灰尘样本中检测到37种FLCMs，总含量为4.33~121.15 ng/g。室内是人的主要活动场所^［13］^，人们在此环境中可能通过手口摄入、皮肤吸收和呼吸吸入含FLCMs的灰尘等途径暴露于FLCMs，从而面临潜在的健康风险。

FLCMs比其他LCMs具有更强的环境持久性、生物累积性和毒性^［14］^。据预测，FLCMs对鱼类、绿藻和水蚤具有急性毒性^［6］^。FLCMs已被发现表现出多种毒性效应。如FLCMs低剂量暴露可增加鲶鱼的抗氧化酶活性^［15］^。FLCMs暴露可导致鸡胚胎干细胞中*CYP1A4*基因表达显著上调和*THRSP*基因表达下调，表明其可能具有潜在的肝毒性，并可能影响脂质代谢和甲状腺分泌^［16］^。此外，FLCMs是过氧化物酶体增殖物激活受体γ的潜在拮抗剂，可能会影响细胞的葡萄糖摄取^［17］^。FLCMs暴露还能破坏斑马鱼的甲状腺激素水平，并通过影响其关键蛋白来扰乱下丘脑-垂体-甲状腺轴^［18］^。Zhang等^［19］^的研究表明，FLCMs可以使受孕大鼠的胎盘发育延迟和黄体酮的释放减少。上述研究表明，FLCMs具有一定的毒性作用。然而，其对人体长期低剂量暴露的健康风险仍缺乏基于真实暴露场景的评估。

随着电子产品的广泛普及，FLCMs的室内暴露问题日益凸显^［12］^。研究表明，由于不同室内场景应用的电子产品有所不同，FLCMs的释放呈现空间异质性，导致其室内赋存浓度出现差异性^［16］^。另外，不同年龄段和性别的人群在生活习惯与生理特性上的差异，可能导致其面临的健康风险不同^［20］^。如蹒跚学步的婴幼儿在地面的爬行行为以及频繁的手口接触，导致其更容易暴露于灰尘中的FLCMs^［21］^。不同年龄段在室内的时长也不同，如学生、公务人员在室内的时间会偏长。《中国人群暴露参数手册》也表明不同性别和年龄人群的暴露参数相差较大^［22］^。如儿童和青少年处于快速生长发育阶段，其呼吸频率、体重等生理参数存在显著个体差异。而老年群体因基础代谢率下降等因素，上述指标普遍低于中青年群体。此外，同年龄段男性在呼吸频率和体重方面通常高于女性，加之不同性别在卫生习惯上的差异，共同导致不同人群在相同室内FLCMs暴露产生差异化的健康风险。因此，解析FLCMs的赋存特征及其不同人群的差异化健康风险，有利于制定更精细化的管控措施，从而为健康风险分级管理提供科学依据。本研究以北京某高校的室内灰尘为研究对象，采用全二维气相色谱-飞行时间质谱（GC×GC-TOF MS）技术同时测定室内灰尘中25种FLCMs的含量，并利用蒙特卡洛模拟评估了FLCMs对不同人群的差异化健康风险。

## 1 实验部分

### 1.1 仪器、试剂与材料

Agilent 7890气相色谱仪（Agilent，美国），Pegasus 4D GC×GC-TOF MS（LECO，美国）。

25种FLCMs标准品购自毕得和阿拉丁公司，纯度均大于98%。25种FLCMs标准品见表1。石英纤维滤膜购自英国沃特曼公司；二氯甲烷和正己烷（均为色谱纯）购自美国Thermo Fisher公司；Milli-Q超纯水（18.2 MΩ·cm）。DMP-d4标准溶液（1 000 ng/mL）购自加拿大TRC公司；考虑到FLCMs稳定同位素内标目前还没有市售，我们选择与FLCMs性质相似的邻苯二甲酸二甲酯-d4（DMP-d4）作为内标。

**表1:** 室内灰尘中FLCMs的检出情况

Compound	Abbreviation	Detection rate/%	Contents/（ng/g）			
			Min	Max	Median	Mean
4-（Difluoro（3，4，5-trifluorophenoxy）methyl）-3，5-difluoro-4′-propyl-1，1′-biphenyl	tFPO-CF2-dF3B	83.3	<LOD	70.3	12.0	11.8
（*trans*，*trans*）-4-（4-Ethoxy-2，3-difluorophenyl）-4′-propyl-1，1′-bi（cyclohexane）	2OdFP3bcH	81.7	<LOD	102	14.3	19.1
*trans，trans*-4′-（4′-Propylbicyclohexyl-4-yl）-3，4，5-trifluorobiphenyl	3bcHtFB	80.0	<LOD	101	13.1	20.3
*trans，trans*-4-Fluorophenyl 4′-propylbicyclohexyl-4-carboxylate	3bcHCaAFP	75.0	<LOD	126	11.1	16.5
1，2-Difluoro-4-［4-（4-pentylcyclohexyl）phenyl］benzene	5cHdFB	56.7	<LOD	187	1.74	12.5
1，2-Difluoro-4-［4-（4-propylcyclohexyl）phenyl］benzene	3cHdFB	53.3	<LOD	299	2.70	20.6
（*trans，trans*）-4-Ethyl-4′-（4-（trifluoromethoxy）phenyl）-1，1′-bi（cyclohexane）	2bcHtFMeOP	50.0	<LOD	31.7	0.220	3.59
2，3-Difluoro-1-methoxy-4-（（*trans*-4-propylcyclohexyl）methoxy）benzene	3cHMeO-MeOdFP	45.0	<LOD	31.5	<LOD	3.87
1-［4-（4-Propylcyclohexyl）cyclohexyl］-4-（trifluoromethoxy）benzene	tFMeO-3bcHP	45.0	<LOD	41.2	<LOD	3.44
5-［4-［4-（4-Ethylcyclohexyl）cyclohexyl］phenyl］-1，2，3-trifluorobenzene	2bcHtFP	41.7	<LOD	4.56	<LOD	0.817
1-Fluoro-4-［4-（4-propylcyclohexyl）phenyl］benzene	3cHFB	36.7	<LOD	15.9	<LOD	1.14
1-Fluoro-4-［4-（4-propylcyclohexyl）cyclohexyl］benzene	3bcHFP	35.0	<LOD	37.6	<LOD	3.66
1-Ethoxy-2，3-difluoro-4-（*trans*-4-pentylcyclohexyl）benzene	5cH2OdFP	25.0	<LOD	13.4	<LOD	1.03
3，4-Difluoro-4′-（4-ethylcyclohexyl）biphenyl	2cHdFB	18.3	<LOD	14.6	<LOD	0.870
1，2-Difluoro-4-［4-（4-pentylcyclohexyl）cyclohexyl］benzene	5bcHdFB	16.7	<LOD	13.1	<LOD	1.14
1，2-Difluoro-4-［4-［4-（4-propylcyclohexyl）cyclohexyl］phenyl］benzene	3bcHdFP	13.3	<LOD	8.64	<LOD	0.389
4-［4-（4-Ethylcyclohexyl）cyclohexyl］-1，2-difluorobenzene	2bcHdFP	11.7	<LOD	3.20	<LOD	0.174
1，2-Difluoro-4-［4-［4-（4-propylcyclohexyl）cyclohexyl］phenyl］benzene	3bcHdFB	10.0	<LOD	7.96	<LOD	0.525
（4-Fluorophenyl） 4-（4-pentylcyclohexyl）cyclohexane-1-carboxylate	5bcHCaAFP	5.0	<LOD	10.3	<LOD	0.386
1，2-Difluoro-4-［4-（4-pentylcyclohexyl）cyclohexyl］benzene	5bcHdFP	1.7	<LOD	2.56	<LOD	0.072
*trans，trans*-4′-Ethyl-4-（3，4，5-trifluorophenyl）bicyclohexyl	2bcHtFP	0	-	-	-	-
1，2-Difluoro-4-［2-fluoro-4-（4-propylphenyl）phenyl］benzene	3tFT	0	-	-	-	-
（4-Cyano-3-fluorophenyl） 4-propylbenzoate	3BzoFCP	0	-	-	-	-
1，2，3-Trifluoro-5-［4-［4-（4-pentylcyclohexyl）cyclohexyl］phenyl］benzene	5bcHtFB	0	-	-	-	-
*trans，trans*-4-（3，4-Difluorophenyl）-4′-vinylbicyclohexyl	dFPVbcH	0	-	-	-	-

### 1.2 标准溶液配制

将标准品溶于正己烷-二氯甲烷（1∶3，v/v）溶液，配制成质量浓度为5 mg/mL的25种FLCMs混合标准储备液；将混合标准储备液用正己烷-二氯甲烷（1∶3，v/v）溶液稀释得到质量浓度为1 000 ng/mL的混合标准应用溶液，并用正己烷-二氯甲烷（1∶3，v/v）溶液配制成质量浓度为0.1、1、10、50、100、200和500 ng/mL的系列标准溶液。将内标标准溶液（1 000 ng/mL）用正己烷-二氯甲烷（1∶3，v/v）溶液进行稀释，配制质量浓度为250 ng/mL的内标标准溶液，于4 ℃冰箱保存备用。

### 1.3 样品采集

2024年4月~7月，在吸尘器金属钢管头部套上尼龙采集袋，用吸尘器吸取北京市某高校校园环境中实验室（*n=*12）、宿舍（*n=*12）、教室（*n=*12）、打印店（*n=*12）和食堂（*n=*12）地面的灰尘。在采集样品时，尽可能地采集各室内场景中的多个位置，包括地面、墙角、桌缝等灰尘聚集的地方。每周采集样品一次，共采集12次。为避免样品污染，采样前将采样袋用二氯甲烷超声清洗2次，然后用超纯水超声清洗1次；每次采样后用甲醇擦拭金属钢管3次。采集的灰尘样品用孔径为150 µm的筛子过筛，并将过筛后的灰尘用铝箔纸包裹好，放入自封袋内，保存在-20 ℃冰箱中，待用。

### 1.4 样品前处理

称取50 mg灰尘放入15 mL玻璃管中，然后加入25 ng内标（DMP-d_4_）。将混合物在摇床上振荡0.5 h。向玻璃管中加入2.5 mL正己烷-二氯甲烷（1∶3，v/v）溶液，涡旋1 min。然后在100 Hz、25 ℃条件下将溶液超声30 min，将超声后的溶液以4 500 r/min的转速离心6 min，收集上清液至15 mL玻璃管中。重复上述操作一次，合并两次上清液。将合并的上清液在氮气流下浓缩至近干，加入200 μL正己烷-二氯甲烷（1∶3，v/v）溶液复溶，溶液经0.22 μm有机滤膜过滤，转移至2 mL样品瓶中，待测。

### 1.5 分析条件

#### 1.5.1 色谱条件

色谱柱：一维色谱柱为Rxi-5MS毛细管柱（30 m×0.25 mm×0.25 μm，Restek，美国）；二维色谱柱为Rxi-17Sil毛细管柱（1.39 m×0.25 mm×0.25 μm，Restek，美国）；载气流速：1.2 mL/min；进样口温度：290 ℃；升温程序：初始温度为100 ℃，保持1 min，以20 ℃/min升至200 ℃；再以3 ℃/min升至300 ℃，保持5 min。二维色谱柱的温度高于一维色谱柱5 ℃，调制器温度高出二维色谱柱25 ℃，调制周期为4 s，冷喷时间为1.2 s，热喷时间为0.8 s。不分流进样，进样量2 µL。

#### 1.5.2 质谱条件

电子轰击离子（EI）源，电离电压70 eV；离子源温度250 °C，传输线温度290 °C；溶剂延迟时间6 min；扫描模式为全扫描，扫描范围为*m/z* 40~480；采集速率：100 spectra/s。其他仪器参数见课题组前期发表的文章^［23］^。

### 1.6 质量保证与质量控制

为了避免实验本底干扰，所有玻璃器皿在使用前均用超纯水和二氯甲烷超声清洗3次，并在100 ℃下干燥8 h。样品处理过程中添加流程空白样品（超纯水），按照与灰尘样品相同的流程进行前处理和检测，用于监测样品处理过程中是否引入污染。样品进行仪器分析前用二氯甲烷清洗系统，减少背景污染。各FLCMs采用7点校正曲线进行定量（0.1、1、10、50、100、200和500 ng/mL），标准曲线的相关系数均大于0.990。向无水硫酸钠中添加标准溶液，用于确定仪器方法的检出限（LOD）和定量限（LOQ）。使用3倍和10倍信噪比（*S/N*）分别确定25种FLCMs的LOD和LOQ，LOD为0.01~0.4 ng/mL，LOQ为0.04~1.34 ng/mL。向无水硫酸钠中添加低、中、高3个不同浓度水平的标准溶液（1、10、100 ng/mL），每个水平重复测定3次，加标回收率为62.1%~139%，相对标准偏差（RSD）为1.13%~28.0%（附表1）。

### 1.7 健康风险评估

室内灰尘是普通人群通过手口摄入、皮肤接触和呼吸吸入等途径接触FLCMs的重要媒介。本研究通过式（1）~（4）评估人体FLCMs的每日摄入量（EDI，ng/（kg·day））^［24-27］^：


$$EDI_{der}=(C\times SA\times DAF\times SAF\times EF)/BW$$ (1)

$$EDI_{ing}=(C\times IngR\times EF)/BW$$ (2)

$$EDI_{inh}=(C\times InhR\times EF)/(BW\times PEF)$$ (3)

$$EDI=EDI_{ing}+EDI_{inh}+EDI_{der}$$ (4)

式中EDI_der_、EDI_ing_、EDI_inh_和EDI分别为经皮肤接触、手口摄入、呼吸吸入和总的灰尘中FLCMs的每日摄入量（ng/（kg·day））；*C*是灰尘中FLCMs的含量（ng/g）；SA代表人的皮肤接触表面积；DAF是皮肤吸收因子（取0.13）^［24］^；SAF是灰尘吸附因子；EF是在室内的暴露频率；BW是平均体重；IngR是摄入率；InhR是吸入率；PEF是颗粒排放因子（1.36×10^6^ m^3^/g）^［24］^。BW、SAF、IngR、InhR、SA和EF的值来自《中国人群暴露参数手册》^［22］^，具体取值见附表2。

**表2:** 各年龄段通过室内灰尘摄入FLCMs的EDI

Age	EDI/（ng/（kg·day））				
	Laboratory	Dormitory	Classroom	Printshop	Canteen
0-1 years （male）	0.927	0.470	0.527	1.587	0.091
0-1 years （female）	0.965	0.489	0.549	1.654	0.095
1-2 years （male）	1.027	0.521	0.584	1.759	0.101
1-2 years （female）	1.069	0.542	0.608	1.831	0.105
2-3 years （male）	0.883	0.447	0.502	1.512	0.087
2-3 years （female）	0.891	0.451	0.506	1.526	0.088
3-6 years （male）	0.839	0.425	0.477	1.436	0.083
3-6 years （female）	0.810	0.411	0.461	1.388	0.080
6-18 years （male）	0.513	0.260	0.292	0.879	0.051
6-18 years （female）	0.499	0.253	0.283	0.854	0.049
18-45 years （male）	0.127	0.064	0.072	0.218	0.013
18-45 years （female）	0.148	0.075	0.084	0.253	0.015
45-60 years （male）	0.126	0.064	0.071	0.215	0.012
45-60 years （female）	0.139	0.070	0.079	0.238	0.014
> 60 years （male）	0.142	0.072	0.081	0.243	0.014
> 60 years （female）	0.158	0.080	0.090	0.271	0.016

利用风险熵（hazard quotient，HQ）计算FLCMs经室内灰尘暴露的健康风险^［12］^：


HQ=EDI/TDI (5)

式中，HQ无量纲，表示FLCMs的非致癌风险；EDI为FLCMs的总摄入量；TDI为每日可容忍摄入量。FLCMs的TDI由无观测不良效应水平（NOAEL）计算得到^［12］^：


TDI=NOAEL/(UF1×UF2×UF3×UF4) (6)

式中，UF1、UF2、UF3和UF4为不确定因子。详见附表3。

危害指数（hazard index，HI）用来评估FLCMs的混合暴露风险^［28］^，计算公式如下：


HI=∑HQ (7)

本研究中对HI的评价基于蒙特卡洛模拟的方法，通过10 000次的迭代使HI结果趋于稳定，将置信水平设为95%，保证风险评估的可靠性。

### 1.8 统计分析

室内灰尘样品中的FLCMs浓度低于LOD时，将其浓度设为LOD/2；低于LOQ但高于LOD的物质按检出水平计算。检出率<40%的FLCMs不纳入统计分析。采用IBM SPSS Statistics 26.0软件进行Mann-Whitney检验，分析各年龄段EDI的差异，斯皮尔曼相关性分析研究FLCMs之间的相关性，*p*<0.05被认为具有统计学意义。使用Crystal Ball 11.1.3.0.000软件进行蒙特卡洛模拟，本文图均为Origin 2024b软件制作。

## 2 结果与讨论

### 2.1 室内灰尘中FLCMs的赋存情况

在60份室内灰尘样品中共检出20种FLCMs，20种FLCMs的总含量范围为3.68~593 ng/g，中位水平为85.1 ng/g。其中，tFPO-CF2-dF3B、2OdFP3bcH、3bcHtFB和3bcHCaAFP的检出率大于70%，3cHMeO-MeOdFP、5cHdFB、3cHdFB、2bcHtFMeOP、2bcHtFP和tFMeO-3bcHP 6种FLCMs的检出率为40%~60%，检出率低于40%的目标物不予讨论（见表1）。室内灰尘中含量最高的FLCMs是2OdFP3bcH，中位水平为14.3 ng/g；其次是3bcHtFB和tFPO-CF2-dF3B，中位水平分别为13.1 ng/g和12.0 ng/g。Liu等^［10］^在美国16个州收集的104个室内灰尘样本中检出了27种FLCMs，占所有LCMs的59%，含量范围为45.3~1 586 ng/g，高于本研究∑FLCMs含量。这可能与电子设备（如手机和电脑）的使用有关，已有研究证明智能手机、电脑等电子设备是室内灰尘中LCMs的主要来源^［10］^。Su等^［16］^在中国南京的室内灰尘样品中检测到12种FLCMs，∑FLCMs含量范围为0.13~2 213 ng/g，高于本研究。这可能与收集的样品来自实验室有关，实验室中大量的电子设备和通风条件使得室内FLCMs的赋存水平较高。Zhu等^［1］^在LCD拆卸车间的灰尘中检出34种FLCMs，总含量水平为225~97 600 ng/g。Cheng等^［11］^在电子垃圾回收工业区的室内灰尘中发现FLCMs占∑LCMs的87.5%，而在室外灰尘样品中只检测到了FLCMs，这与FLCMs具有更强的持久性以及FLCMs是电子产品中主导地位的LCMs有关。Yang等^［12］^在中国北京的室内灰尘样品中检测到37种FLCMs，∑FLCMs含量范围为4.33~121.15 ng/g，与本研究含量水平一致。这些结果表明，FLCMs的含量存在地理位置上的差异，不同场景下室内灰尘中的FLCMs也有所不同。这可能与电子设备的数量、大小以及清洁频率有关，因为电子产品是室内灰尘中FLCMs的主要来源^［29］^。

### 2.2 不同室内场景下FLCMs的分布特征

不同室内场景的总FLCMs含量相差较大，打印店的室内灰尘中FLCMs的含量最高，∑FLCMs为206~593 ng/g；其次是实验室（89.0~219 ng/g）、教室（28.4~137 ng/g）、宿舍（39.2~95.7 ng/g）和食堂（3.68~47.6 ng/g）。Su等^［16］^在中国南京不同室内场景下检测到的∑LCMs也存在类似的差异：实验室>教室>宿舍>食堂。这种现象可能与各室内场景不同的电子设备数量与种类相关。如打印店普遍存在激光打印设备及电脑显示屏，并且在打印机工作时产生的高温也会加速FCLMs向室内灰尘的迁移释放。Hoang等^［30］^和Khan等^［31］^也发现电子产品数量较多的室内灰尘样本中的有机污染物含量高于普通住宅。实验室的设备老化，并且通风效果差，使得FCLMs在室内灰尘累积。另外，清洁频率对室内灰尘中的污染物也有显著影响^［32］^。宿舍和食堂的清洁比打印店等更加频繁，不利于FLCMs的积累，故其含量较低。电子产品的使用导致FLCMs在室内灰尘中的赋存，而电子产品（手机和电脑等）已成为人们生活中不可或缺的物品，因此有必要评估其对人体的健康风险。

通过分析不同室内场景灰尘样品中FLCMs的组成特征，发现其分布具有空间异质性（图1），这可能与不同室内场景的电子设备使用特征及人类活动相关。从组分构成来看，实验室灰尘中的主要污染物是3bcHtFB，占比30%，其次为2OdFP3bcH（23%）和3bcHCaAFP（15%），这种组成特征可能与实验器材中特定的电子显示屏（如电脑）有关。宿舍灰尘中以2OdFP3bcH（29%）、tFPO-CF2-dF3B（19%）和3bcHCaAFP（18%）为主要污染物，这可能与手机和平板电脑的使用相关。教室灰尘则以3bcHtFB（21%）、tFPO-CF2-dF3B（17%）和3cHdFB（15%）为主，其组成可能与手机以及教室的多媒体设备密切相关。打印店灰尘中的FLCMs主要为3cHdFB（29%）、5cHdFB（18%）和3bcHCaAFP（14%）；值得注意的是，打印店灰尘样品中3cHdFB显著高于其他场所，这可能与打印设备的电子显示屏有关。食堂灰尘中的FLCMs主要为2OdFP3bcH（30%）、tFMeO-3bcHP（20%）和3bcHtFB（17%），这可能与食堂的清洁频率有关，研究表明清洁频率可能会影响FLCMs赋存水平^［24］^。这些不同的电子设备导致了FLCMs在各室内场景的分布差异，研究表明不同电子设备中FLCMs的差异很大，如手机中的2OdFP3bcH含量高于计算机中的含量^［11］^。另外，不同品牌的电脑或手机以及样品的采样时间和范围也可能影响各场景的FLCMs分布水平^［21］^。

**图1:**
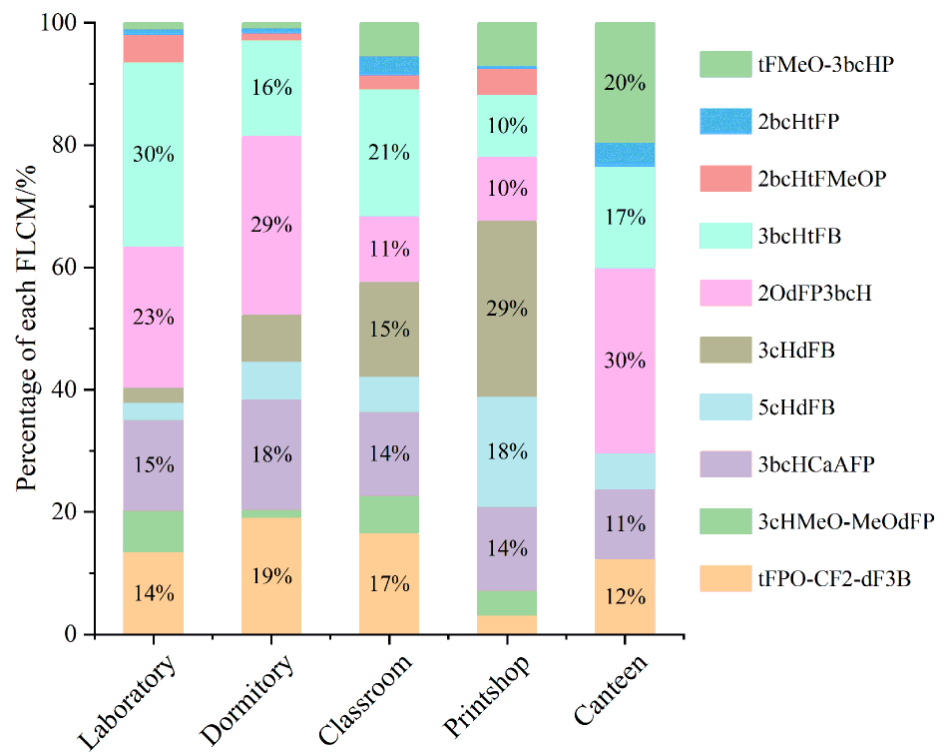
不同室内场景灰尘中各FLCM的占比

### 2.3 FLCMs各物质间的相关性分析

利用斯皮尔曼秩相关分析法，计算了室内灰尘中FLCMs的相关性，用以识别灰尘中FLCMs的潜在污染源，计算结果如图2所示。室内灰尘中2bcHtFP和2bcHtFMeOP具有显著的负相关关系（*r*=-0.829，*p*<0.05），说明二者的来源可能不同。在*p*<0.05（双尾）条件下，3cHdFB和5cHdFB（*r*=0.722）、3cHdFB和tFMeO-3bcHP（*r*=0.676）、3cHdFB和2bcHtFMeOP（*r*=0.601）、5cHdFB和2bcHtFMeOP（*r*=0.539）表明出显著的正相关关系，表明这些化合物可能来自相同的污染源。这与Liu等^［10］^报道的室内灰尘中的FLCMs主要来源于智能手机一致。

**图2:**
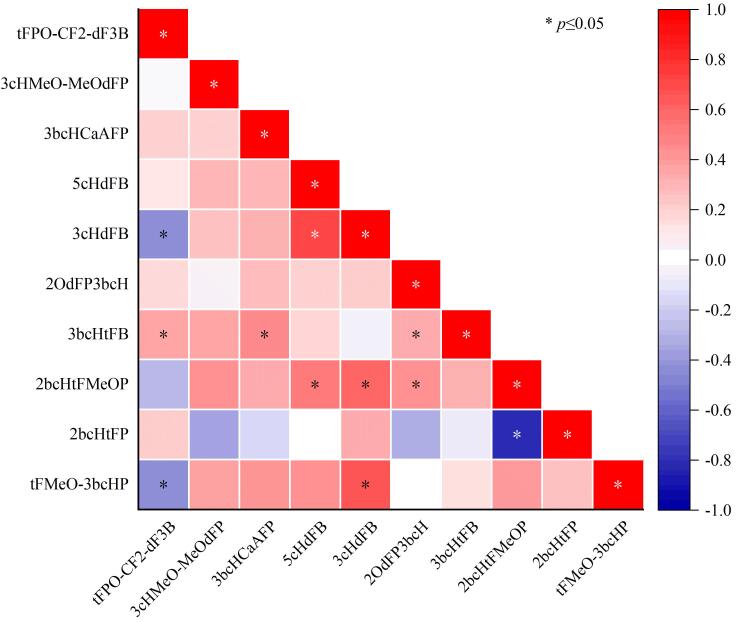
各FLCM间的相关性

### 2.4 健康风险评估

利用测得的室内灰尘中FLCMs的含量，评估了不同年龄段人群通过摄入、皮肤接触和吸入方式对FLCMs的暴露。表2列出了不同室内场景下各年龄段的∑FLCMs中位摄入水平，由于∑FLCMs的含量水平的排序为打印店>实验室>教室>宿舍>食堂，因此导致相应的高摄入量。对各年龄段的EDI进行了差异化分析，发现0~1和1~2岁年龄段的EDI显著高于6~18、18~45、45~60和>60这4个年龄段的EDI（Mann-Whitney U检验，*p*<0.001）；2~3和3~6岁年龄段的EDI显著高于6~18、18~45、45~60和>60这4个年龄段的EDI（*p*<0.05）；6~18岁年龄段的EDI与18~45、45~60和>60这3个年龄段的EDI有显著差异（*p*<0.05）。这些差异表明，未成年人群对室内灰尘FLCMs暴露的敏感性更高，这与之前的研究一致^［24］^。需要特别强调的是，0~18岁和>60岁的群体的EDI值主要是基于暴露参数和生理行为特征的理论推算结果，但他们在高校环境中的实际活动较少。而18~45岁、45~60岁是高校环境的主要活动群体，虽然年龄段的暴露风险低于上述敏感人群，但相对长时间的暴露所产生的相关健康风险仍不容忽视。另外，发现该群体中女性的暴露风险略高于男性（表2），这可能是由于女性通常在室内的时间较长，暴露频率更高导致的。总的来说，在高校环境中，一些特殊场所的人群（如打印店的工作人员）具有相对较高的EDI；需要关注这些人群接触FLCMs的健康风险。

为了评估单个FLCM的非致癌风险，计算了高校群体（18~45岁）的HQ，以评估FLCMs的非致癌健康风险。基于中位水平暴露情景的HQ计算结果显示（附表4），tFPO-CF2-dF3B和2bcHtFMeOP的HQ值较高（>1.00×10^-6^），需要关注其潜在的暴露风险。具体而言，tFPO-CF2-dF3B在实验室、宿舍、教室均有较高的HQ；2bcHtFMeOP则在实验室和打印店具有较高的HQ。另外，3cHdFB、tFMeO-3bcHP在打印店具有较高的HQ；3bcHtFB在实验室展现出较高的HQ。不同室内场景的FLCMs风险特征差异与FLCMs的含量以及毒性密切相关。如tFPO-CF2-dF3B在实验室灰尘的含量较低（中位数：16.20 ng/g），但其毒性较高（TDI：6 504 ng/（kg·day）），导致其非致癌风险较高（男：2.36×10^-6^，女：2.75×10^-6^）。

图3列出了不同室内场景下各年龄段HI的概率分布，对比不同室内场景的各年龄阶段的非致癌风险可知，打印店对各年龄段人群的非致癌风险均表现出较高水平（HI中位数范围：1.88×10^-5^~1.60×10^-4^）。与其他场所相比，处于打印店场所的人群其健康风险HI值高出约2~6倍。而同一室内场景下的各年龄段的性别导致的非致癌风险差异相对较小。具体来说，女性的非致癌风险略高于男性，导致这一现象的原因可能是女性的体重小于男性，且女性的暴露周期和日均暴露时间高于男性。另外，本研究样本采集自高校校园，不同室内场景的使用群体存在差异，需结合具体场景分析高暴露人群特征。如打印店的非致癌风险最高（HI中位数，男：1.90×10^-5^，女：2.21×10^-5^），这与打印店中电脑和打印机显示器的密集使用密切相关。Liu等^［10］^的研究表明，室内灰尘中的FLCMs主要来自智能手机和电脑显示屏，而打印店作为学生和教师频繁打印材料的场所，其电子设备的高频运行可能加速FLCMs的释放与累积。因此，长期驻留的打印店工作人员及频繁出入的学生群体的健康风险需重点关注。实验室的非致癌风险（男：9.31×10^-6^，女：1.08×10^-5^）高于宿舍（男：3.87×10^-6^，女：4.50×10^-6^）和教室（男：5.25×10^-6^，女：6.10×10^-6^），这与Su等^［16］^的关于实验室FLCMs的赋存研究一致。这可能与实验室内电子设备（如电脑、仪器显示屏）密集且通风条件有限有关，科研人员长期在此实验室工作，需关注其由FLCMs导致的健康风险。而食堂由于停留时间较短及清洁频繁，其非致癌风险较低。总体而言，不同场景的各年龄阶段的HI均小于1，表明以室内灰尘为介质的FLCMs暴露无明显的非致癌风险。

**图3:**
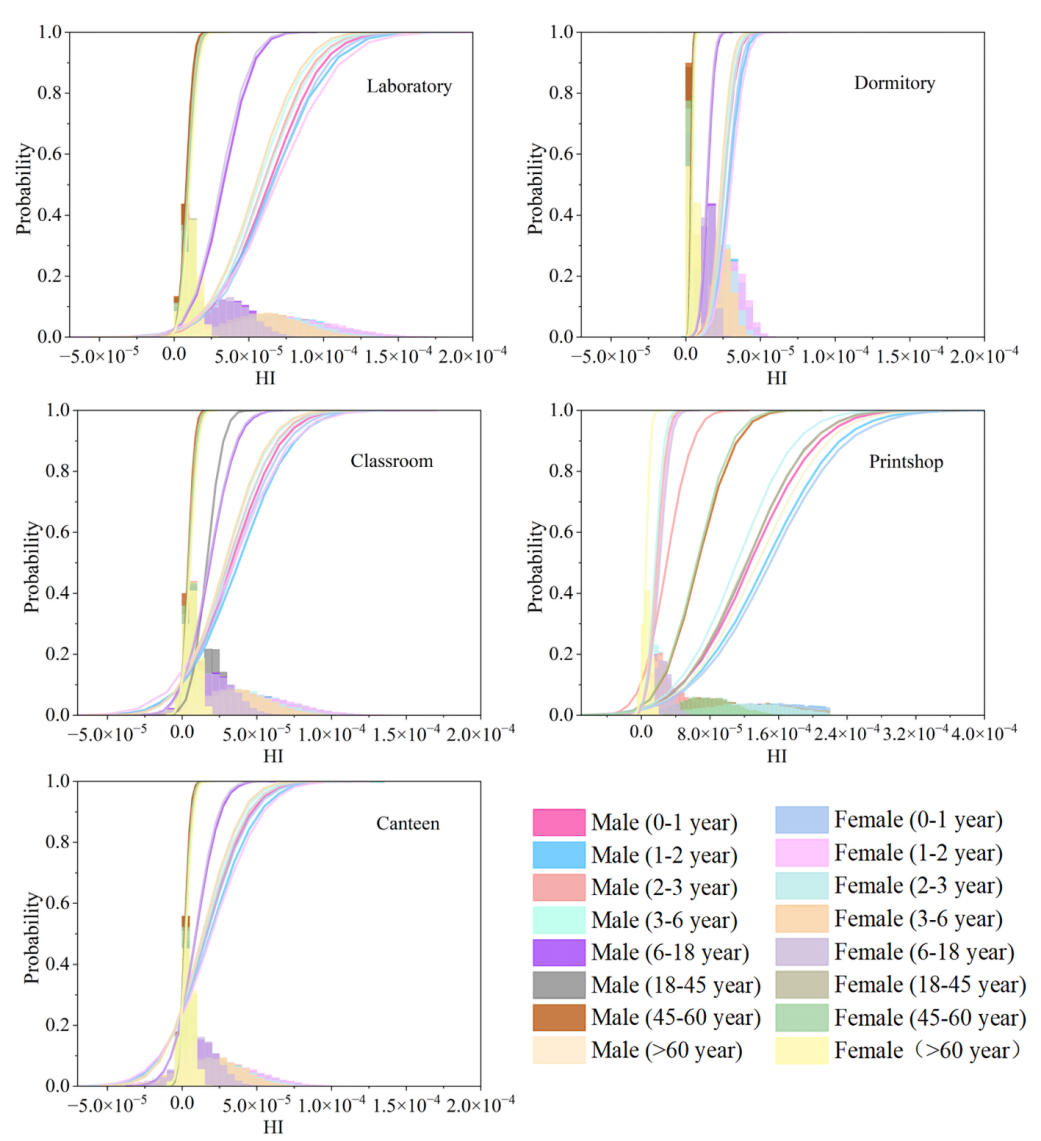
基于蒙特卡洛模拟的室内灰尘中FLCMs对不同人群的非致癌风险概率分布

## 3 结论

本研究揭示了不同室内场景灰尘中FLCMs的赋存特征及健康风险差异。发现2OdFP3bcH、3bcHtFB和tFPO-CF2-dF3B为灰尘中主要的污染物。不同室内场景中的FLCMs分布差异主要与电子产品的数量以及类型有关。另外，清洁频率也是影响室内灰尘中FLCMs分布的重要因素。本研究利用HQ和蒙特卡洛模拟评估了高校环境中FLCMs的非致癌风险。结果显示，不同室内场景和不同人群的非致癌风险存在差异。另外，还发现高校主要群体（18~45岁和45~60岁）中女性的非致癌风险更高。人群差异化的非致癌风险与呼吸频率、体重等生理参数及生活习惯密切相关。本研究首次通过多场景、多人群暴露模型阐明了FLCMs的室内污染特征与风险分布规律，为制定基于敏感人群的差异化暴露防控策略提供了数据支撑。
